# Hemodynamic modeling of aortic arch aneurysm treatment using the Castor™ branched stent graft: a virtual coil embolization simulation framework

**DOI:** 10.3389/fphys.2025.1629346

**Published:** 2025-07-02

**Authors:** Xiaoqiang Ye, Jianning Zhang, Zhiguo Cheng, Fenghui Lu, Zijing Wen, Guanghao Yu, Guangxin Chen, Fengjie Xie, Dan Qiao, Jian Xing, Wenchang Tan, Dongliang Zhao, Mingming Ren

**Affiliations:** ^1^ Department of Cardiovascular Surgery, Peking University Shenzhen Hospital, Shenzhen, Guangdong, China; ^2^ School of Life Sciences, Mudanjiang Medical University, Mudanjiang, Heilongjiang, China; ^3^ Shenzhen Bay Laboratory, Shenzhen, Guangdong, China; ^4^ Medical Image College, Mudanjiang Medical University, Mudanjiang, Heilongjiang, China; ^5^ Department of Critical Care Medicine, Hongqi Hospital Affiliated to Mudanjiang Medical University, Mudanjiang, Heilongjiang, China; ^6^ Department of Pathology, Sir Run Run Shaw Hospital, Zhejiang University, Hangzhou, Zhejiang, China; ^7^ Department of Mechanics and Engineering Science, College of Engineering, Peking University, Beijing, China

**Keywords:** aortic arch aneurysm, Castor^TM^ single-branched stent graft, hemodynamic modeling, virtual coil embolization simulation framework, 3-element windkessel

## Abstract

**Introduction:**

Aortic arch aneurysm (AAA) refers to pathological dilation of the aortic arch, carrying high rupture risks under hypertensive conditions with critical mortality rates, thus remaining a key research focus. The Castor™ single-branched stent-graft effectively isolates the aneurysm from circulatory pressure and is clinically combined with coil embolization to enhance therapeutic outcomes. However, comprehensive hemodynamic analyses evaluating the therapeutic efficacy of this combined approach remain lacking.

**Methods:**

This study establishes the first patient-specific hemodynamic model for Castor™ stent-graft-treated AAA, investigating pre- and postoperative biomechanical changes. A novel virtual coil embolization simulation methodology was developed to analyze the effects of coil length and morphology on aneurysmal hemodynamics.

**Results and discussion:**

Results demonstrate significant aneurysmal pressure attenuation post-stent implantation, complete endoleak prevention, and enhanced proximal left common carotid (LCC) flow, while coil embolization induces localized hemodynamic alterations without perturbing major branch outflow. Progressive coil lengthening and packing density elevation correlate with expanded low-TAWSS regions and elevated OSI/RRT values, mechanistically confirming thrombosis acceleration. Systematic evaluation reveals synergistic hemodynamic interplay between stent-graft-mediated macroscale flow reconstruction and coil-induced thrombogenic microenvironments, providing critical insights for personalized AAA management.

## 1 Introduction

The aortic arch aneurysm (AAA) is defined as a pathological dilation of the aortic arch segment that frequently involves its major branch vessels, including the brachiocephalic trunk, left common carotid artery, and left subclavian artery (Rong et al., 2022). The pathogen of AAA shares common pathological mechanisms with ascending aortic aneurysms, with major risk factors including hypertension, atherosclerosis, and genetic connective tissue disorders such as Marfan syndrome (Liang et al., 2022; Li et al., 2024; Zhang et al., 2024). Epidemiological studies indicate that AAA account for approximately 10% of all thoracic aortic aneurysms (Nana et al., 2024). The clinical significance of AAA is underscored by their high mortality risk when left untreated, with rupture-associated mortality rates ranging from 42% to 74% as reported in multiple landmark studies (Crawford and DeNatale, 1986; Perko et al., 1995; Steinlauf et al., 2021). Current therapeutic approaches have evolved to include three principal modalities: open surgical repair, hybrid debranching with endovascular repair, and total endovascular repair (Qiao et al., 2019; Gelpi et al., 2021). Hemodynamic characterization remains a critical research focus in AAA management, particularly in understanding intraluminal flow dynamics, computational modeling of various endovascular interventions, and experimental validation of surgical outcomes. These investigations aim to optimize treatment strategies and improve patient prognosis.

The Castor™ branched stent graft represents an innovative integrated device for treating aortic arch pathologies, including aortic arch aneurysms and dissections. This novel design preserves blood flow to arch branch vessels (particularly the left subclavian artery, LSA) while minimizing complications associated with conventional thoracic endovascular aortic repair (TEVAR), such as endoleaks and stent migration (Yao et al., 2022). Compared to traditional chimney/fenestration techniques, the integrated branched configuration significantly reduces gutter-related endoleaks and improves hemodynamic stability. The tapered design (proximal 36 mm to distal 26 mm) better conforms to native aortic anatomy, thereby reducing flow disturbances and enhancing device performance (Yao et al., 2022). Clinical applications of the Castor™ single-branched stent graft have expanded to include: aortic arch aneurysms (Ren et al., 2024), penetrating aortic ulcers (Yao et al., 2022), type B aortic dissections (Jing et al., 2020; Wu et al., 2024). Complex cases involving Kommerell’s diverticulum with left aberrant subclavian artery stenosis (Rodriguez-Perez et al., 2023). In type B aortic dissection management, the Castor™ device demonstrates particular efficacy by reducing false lumen pressure and promoting favorable aortic remodeling. The unique 5-mm retrograde branch design optimizes the proximal landing zone while maintaining LSA patency, which may decrease postoperative stroke risk by preserving critical cerebrovascular circulation (Nana et al., 2024).

Coil embolization technology, while primarily established as a cornerstone of intracranial aneurysm treatment, has demonstrated significant progress in material science, technical optimization, and clinical outcomes that have enabled its successful translation to aortic interventions (Karadeli and Kuram, 2024). The conventional Guglielmi detachable coil system has been progressively supplanted by next-generation modified coils, exemplified by hydrogel-expandable coils whose water-swelling properties enhance packing density and reduce long-term recanalization rates. In the context of TEVAR, adjunctive coil embolization has emerged as an effective strategy for managing endoleaks by reducing persistent false lumen perfusion, preventing aortic expansion, and promoting false lumen thrombosis (Qiao et al., 2020). For complex aortic arch pathologies including dissections and aneurysms, coil embolization of critical branch vessels has proven valuable in mitigating endoleak risk when combined with chimney or fenestrated stent techniques, thereby improving overall procedural success rates (Rakestraw et al., 2020). Despite these clinical advances, a notable research gap persists regarding hemodynamic analyses to optimize coil packing strategies (Ahmed et al., 2014). Such investigations could provide critical insights for minimizing turbulent flow patterns and residual perfusion, potentially enhancing the therapeutic efficacy of aortic coil embolization procedures.

Hemodynamics has been utilized to characterize blood flow patterns in AAA (Tan et al., 2009), assess thrombus formation (Sengupta et al., 2022a), and evaluate the risk of adverse events (Ferrer et al., 2024; Fan et al., 2025). During TEVAR, branched stent-grafts are employed to exclude AAA while simultaneously establishing a blood flow conduit between LSA and the descending aorta (DA) (Van Bakel et al., 2018). Fluid-structure interaction (FSI) analysis of the branched modular stent-graft for AAA treatment demonstrated that the device minimally altered arterial wall dynamics and exhibited low susceptibility to migration and endoleak (Da Silva et al., 2023). Computational fluid dynamics (CFD) studies on the double-branched endograft for AAA repair revealed that it significantly altered aortic flow patterns, resulting in increased spatial variation of wall shear stress in the ascending aorta and the aortic arch (Zhu et al., 2019). Another CFD study indicated that the double-branched endograft ensured sufficient blood perfusion to the supra-aortic branches and restored flow patterns comparable to those observed in healthy aortas (Sengupta et al., 2022b). Finite element analysis (FEA) combined with hemodynamic simulations of the customized stent-graft for AAA management enabled the identification of regions at high risk of aortic wall failure based on stress distribution (Bologna et al., 2023). Hemodynamic assessment of the Nexus™ device for AAA intervention confirmed its technical success in restoring normal flow patterns within the aortic arch (Sengupta et al., 2023). Although the Castor™ single-branched stent graft device has become an important modality for complex aortic arch repair, detailed hemodynamic characterization of this specific device remains lacking.

**FIGURE 1:**
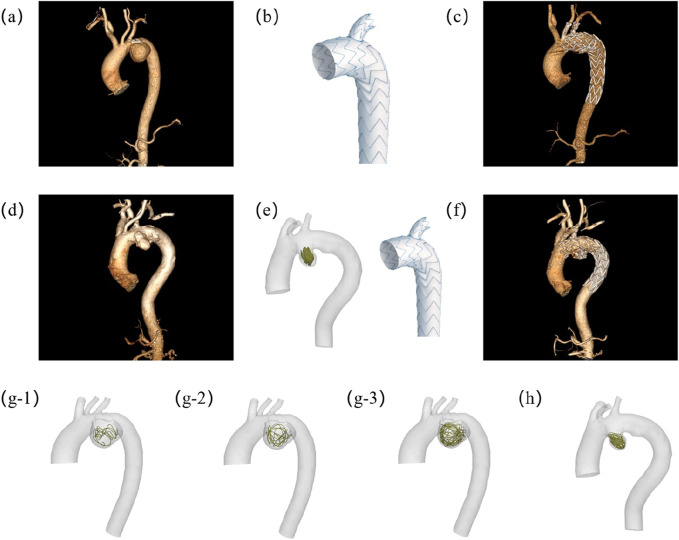
Experimental design schematic demonstrating: **(a)** Preoperative vascular reconstruction of Patient-1; **(b)** Castor™ single-branched stent-graft configuration; **(c)** Postoperative hemodynamic reconstruction in Patient-1; **(d)** Baseline anatomical modeling of Patient-2; **(e)** Integrated 2D coil deployment with branched endograft implantation in Patient-2; **(f)** Post-interventional flow domain visualization of Patient-2; **(g)** Multi-length coil embolization simulation model for Patient-1; **(h)** 3D coil embolization simulation model for Patient-2. The lengths of the coils in (g1), (g2), and (g3) are 40 cm, 80 cm and 160 cm respectively. (g1) is 40 cm.

**FIGURE 2:**
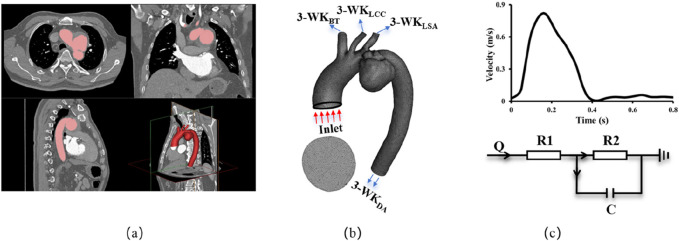
Methodology schematics illustrating **(a)** 3D anatomical reconstruction based on CTA; **(b)** Finite Volume Method mesh generation with outlet nomenclature, BT: Brachiocephalic Trunk, LCC: Left Common Carotid, LSA: Left Subclavian Artery, DA: Descending Aorta; **(c)** Implementation of velocity boundary conditions at the inlet and 3-element windkessel model boundary conditions at the outlets.

**FIGURE 3:**
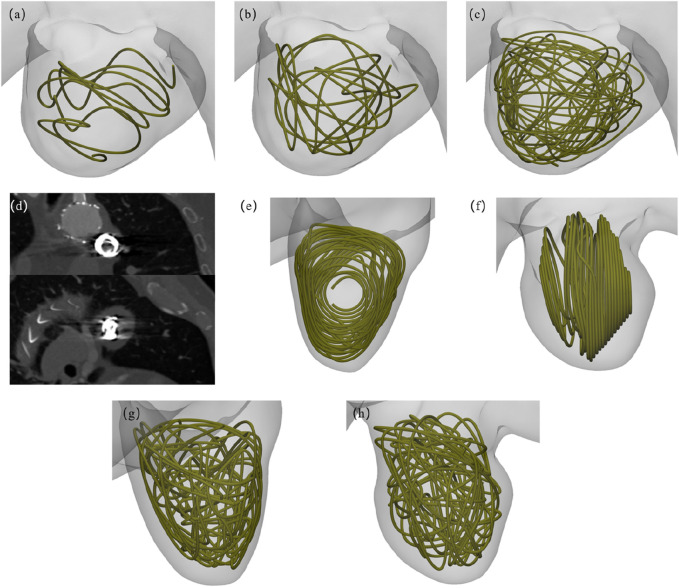
Virtual coil embolization intervention schematics: **(a–c)** Multi-length coil deployment configurations in Patient-1; **(d)** Biplanar projections from postoperative 2D computed tomography angiography (CTA) of Patient-2; **(e, f)** Dual-view 2D embolization modeling reconstructions in Patient-2; **(g, h)** Orthogonal perspective 3D coil packing simulations in Patient-2.

Hemodynamic assessment serves dual critical roles in aneurysm management: providing essential blood flow characteristics and enabling virtual evaluation of surgical/interventional procedures. This study focuses on AAA, conducting comparative hemodynamic analyses of two treatment approaches: 1) standalone Castor™ single-branched stent graft implantation *versus* (Figures 1a–c). 2) combined Castor™ stent graft with coil embolization (Figures 1d–f). The investigation encompasses pre- and post-interventional hemodynamic states for both therapeutic strategies. A unique aspect of this research involves performing virtual coil embolization procedures on patients who underwent Castor™ stent graft placement alone, allowing systematic exploration of hemodynamic field variations associated with different coil packing densities (Figures 1(g-1,g-2,g-3,h)). Key hemodynamic parameters including pressure distribution, velocity fields, time average wall shear stress (TAWSS), oscillatory shear index (OSI), and flow rates were quantitatively analyzed to assess treatment outcomes. The comprehensive study framework is shown in Figure 1.

## 2 Materials and methods

### 2.1 Study design

This retrospective study aimed to investigate the hemodynamic mechanisms of competitive flow between Castor™ single-branched stent grafts with and without adjunctive coil embolization in AAA treatment. The study cohort included two patients who underwent computed tomography angiography (CTA) of the AAA both preoperatively and following surgical (∼1 month) revascularization. AAA Patient-1 underwent endovascular repair using a Castor™ single-branched stent graft (MicroPort Medical Co., Ltd., China) alone, while AAA Patient-2 received combined Castor™ single-branched stent grafts deployment with adjunctive 2D coil (Boston Scientific, US) embolization. For virtual simulation protocols, three coils of varying lengths were digitally implanted within the aneurysm sac of AAA Patient-1 to evaluate length-dependent hemodynamic effects. In contrast, a 3D helical coil embolization was modeled in AAA Patient-2 to investigate the influence of geometric complexity on flow patterns. This comparative framework enabled a comprehensive hemodynamic analysis of both coil length parameters and morphological features (2D vs. 3D spatial distribution). The study protocol received approval from the Institutional Review Board (IRB) of Shenzhen Bay Laboratory, and all participants provided written informed consent. Pre- and post-operative computed tomography angiography (CTA) images from two patients were utilized for computational hemodynamic modeling. Additionally, 5 virtual embolization procedures using coils were simulated. In total, 9 complex hemodynamic models were constructed and analyzed in this study (Figures 1a–c).

### 2.2 Imaging acquisition

Similar to the previous studies (Zhao et al., 2020; Liu et al., 2022), all patient scans were acquired using a dual-source CT scanner (Siemens Definition, Forchheim, Germany) under controlled heart rate conditions (≤65 bpm). Following an initial non-contrast survey scan, contrast-enhanced CTA was performed using Iopromide (Ultravist 370, Bayer Healthcare, Morristown, United States) administered at 1.0 mL/kg body weight, injected at 5 mL/s, followed by a 50 mL saline flush at the same rate. The imaging protocol employed the following parameters: Detector configuration: 2 × 64 × 0.6 mm collimation, Tube settings: 120 kVp, tube current modulated by patient body habitus, Gantry rotation time: 330 ms, Pitch: 0.2–0.43 (automatically adjusted based on heart rate). This configuration enabled simultaneous multi-planar cross-sectional imaging, allowing visualization of the AAA and Castor™ single-branched stent grafts within a single breath-hold. Images were reconstructed with a slice thickness/increment of 0.7/0.4 mm, using a B26f kernel at a temporal resolution of 83 ms (half-scan reconstruction).

### 2.3 Geometrical models

As shown in Figures 2a, 3d gemometrical reconstruction of AAA and Castor™ single-branched stent grafts were extracted from CTA patient images using 3D slicer software. The blood flow area was meshed using ANSYS ICEM (ANSYS Inc., Canonsburg, United States) shown in Figure 2b. A mesh dependency was conducted such that the relative error in two consecutive mesh refinements was <1% for the maximum velocity of steady state flow with inlet flow velocity equal to the time-averaged velocity over a cardiac cycle. A mesh independence study was conducted for Patient-1 using CFD with tetrahedral volume elements. Models comprising approximately 300,000, 1,000,000, 3,000,000, and 6,000,000 elements were compared. As shown in Supplementary Figure S1, the pressure differences at the outlets between the 3,000,000 element and 6,000,000 element meshes were negligible (<0.005%). However, the computational resource requirement for the approximately 6,000,000-element model (230.79 core-hours) was 1.87 times greater than that for the approximately 3,000,000-element model. Based on this mesh independence analysis, the four pre- and post-operative models for both patients were meshed with approximately 3,000,000 tetrahedral elements (element size range: 0.4–0.8 mm). The five models incorporating virtual coils required higher mesh resolution and were therefore discretized with approximately 6,000,000 tetrahedral elements (element size range: 0.2–0.6 mm).

### 2.4 Virtual coil embolization protocol

The virtual embolization procedure employed platinum coils with a standardized diameter of 0.6 mm, It was placed within the aneurysm region as shown in Figure 3. Geometric primitives representing coil configurations were generated within predefined aneurysm regions using Rhinoceros® 7.0 (Robert McNeel & Associates) parametric modeling, where non-overlapping and non-intersecting constraints were rigorously enforced during coil packing simulations. For the virtual embolization procedures, the coil dimensions were strictly based on the Boston Scientific coils used in Patient-2, featuring an overall diameter of 18 mm and a length of 40 cm. Three virtual coil embolization procedures were performed for Patient-1, employing coils of lengths 40 cm, 80 cm, and 160 cm, respectively. Patient-specific configurations were implemented as follows: AAA Patient-1 received different lengths coils, displayed in Figures 3a–c, while AAA Patient-2 consists of a 2D visualization and a 3D spiral coil, displayed in Figures 3e–h. Animations illustrating the virtual embolization procedures for AAA using the different coils are provided in the Supplementary Video.

### 2.5 3D computational model

The Navier-Stokes and continuity equations were solved using the commercial software solver FLUENT (ANSYS, Inc., Canonsburg, United States) when the AAA and coronary arteries were assumed to be rigid and impermeable. Four cardiac cycles were required to achieve convergence for the transient analysis similar to the previous studies (Liu et al., 2022; Yu et al., 2025). A constant time step was employed, where ∆t = 0.01 s with 84 total time step per cardiac cycle. Although blood is a suspension of particles, it behaves as a Newtonian fluid in vessels with diameters >1 mm. The viscosity (μ) and density (ρ) of the solution were assumed as 3.5 × 10^−3^ Pa·s and 1060 kg/m^3^, respectively, to mimic blood flow with a hematocrit of about 45% in these arteries. The measured pressure wave in Figure 2c was set as the boundary condition at the inlet of AAA. The 3-element windkessel resistance boundary condition was assigned to each outlet. Hemodynamic parameters including time-averaged (over a cardiac cycle) WSS and OSI were determined from the computed flow fields.

In the 3-element windkessel, the total resistance of the vessel $R_{Total}$ is given by the following equation:

$$R_{Total}=\frac{P_{m}-P_{out}}{{\bar{Q}}_{in}}$$

$$P_{m}=P_{d}+\frac{1}{3}\left(P_{s}-P_{d}\right)$$

where $P_{s}$ is the patient’s systolic blood pressure, $P_{d}$ is the patient’s diastolic blood pressure, ${\bar{Q}}_{in}$ is the total arterial flow. $P_{out}$ is the capillary pressure, $P_{out}=4426\;Pa$ . (Parazynski et al., 1993).

Based on the parallel resistance principle, the total equivalent resistance at the *i*th outlet ( $R_{Total}^{i}$ ) can be derived as $R_{Total}^{i}=\frac{A_{Total}}{A^{i}}R_{Total}$ , where $A_{Total}$ denotes the total cross-sectional area of all parallel branches, $A^{i}$ represents the cross-sectional area of the *i*th branch. Additionally, the proximal resistance at the *i*th outlet ( $R_{1}^{i}$ ) was defined as the impedance between the upstream node and the branch bifurcation point.

$$R_{1}^{i}=\frac{\rho_{f}c_{d}^{i}}{A_{d}^{i}}$$

where $c_{d}^{i}=\xi {D_{d}^{i}}^{-0.5}$ , $\xi =0.72m^{\frac{2}{3}}s^{-1}$ , $D_{d}^{i}$ is the diastolic diameter of the *i*th outlet, measured at peak relaxation phase. (Alastruey et al., 2009).

The distal resistance of the *i*th outlet ( $R_{2}^{i}$ ) was calculated as: $R_{2}^{i}=R_{Total}^{i}-R_{1}^{i}$

The global vascular compliance (*C*_*T*_), quantifying the systemic energy storage capacity, was determined via:

$$C_{T}=\frac{Q_{\max}-Q_{\min}}{P_{s}-P_{d}}\Delta t$$

where $Q_{\max}$ and $Q_{\min}$ mean maximum and minimum flow velocities derived from aortic root inflow waveform, Δt : time interva between $Q_{\max}$ and $Q_{\min}$ occurrence. $P_{s}$ and $P_{d}$ means systolic and diastolic pressures averaged over the global arterial pressure waveform. This formulation integrates flow dynamics and pressure-volume relationships to characterize arterial buffering function.

### 2.6 Statistical analysis

The mean ± SE (standard error) of morphometric and hemodynamic parameters were computed by averaging over all subjects in each group. The student t-test (GraphPad Prism 5 software) was used to compare these parameters, where p value <0.05 represented a statistically significant difference.

## 3 Results

Hemodynamic simulations were conducted using patient-specific boundary conditions parameterized by a 3-element windkessel model, with detailed computational parameters summarized in Table 1. The hemodynamic profiles of AAA patients were systematically evaluated, comparing preoperative conditions to postoperative outcomes following endovascular repair with Castor™ single-branched stent grafts. Hemodynamic simulations yielded pressure values that demonstrated good agreement with clinically measured blood pressure data. For Patient-1, the pre-operative measured systolic/diastolic blood pressures were 117/82 mmHg, while the corresponding simulated pressures were 118.27/82.69 mmHg. Similarly, for Patient-2, the pre-operative measured pressures were 124/87 mmHg, and the simulated pressures were 125.85/88.31 mmHg. The overall computational errors ranged from 0.84% to 1.51%, indicating that the CFD results accurately reflect the real hemodynamic environment. Furthermore, virtual embolization simulations of coil deployment were performed, and hemodynamic characteristics under varying coil configurations were comprehensively analyzed.

**TABLE 1:** Boundary conditions in the 3-element windkessel model were individualized based on patient specific hemodynamic data.

	Patient-1				Patient-2			
Outlet parameters	BT	LCC	LSA	DA	BT	LCC	LSA	DA
R1(Pa·s/m^3^)	2.82E+07	9.17E+07	7.28E+07	7.72E+06	7.605E+06	2.800E+06	2.806E+06	1.943E+05
R2(Pa·s/m^3^)	1.88E+08	3.97E+08	3.45E+08	5.60E+07	3.814E+08	1.263E+09	8.201E+08	1.560E+08
C (m^3^/Pa)	1.41E-06	1.72E-06	1.64E-06	1.18E-06	2.278E-07	5.239E-07	5.513E-07	5.019E-07

**FIGURE 4:**
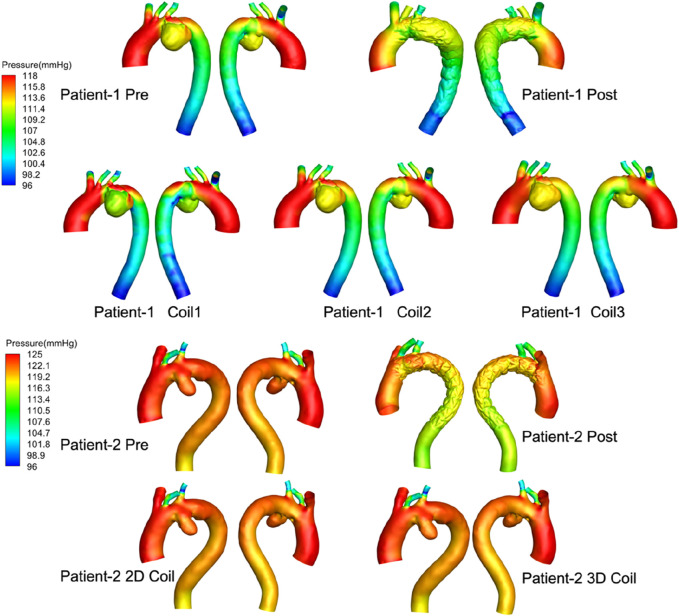
The pressure distribution profiles at end-diastolic all computational frameworks. Computer-generated 3D models of aortic aneurysms for two patients, labeled “Patient-1” and “Patient-2.” Each row shows arterial pressure distributions in millimeters of mercury (mmHg) with variations from pre-treatment, post-treatment, and different coil interventions.

Pressure: As shown in Figure 4 through end-diastolic pressure distribution contours: Patient-1 exhibited aortic pressure gradients ranging 118–96 mmHg, while Patient-2 demonstrated elevated pressure magnitudes (125–96 mmHg). Comparative analysis revealed pressure gradient attenuation following stent-graft deployment, with statistically significant reduction in Patient-2 (p < 0.05). Post-embolization hemodynamic patterns in AAA domains manifested progressive elevation in mural stress distribution correlating with coil length and 3D coil packing complexity, indicating aneurysmal wall stress redistribution mechanisms.

**FIGURE 5:**
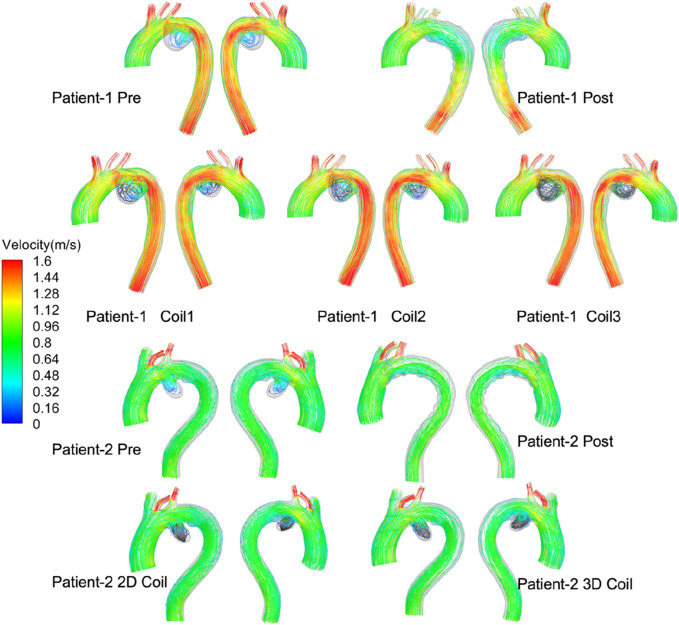
The velocity stream at end-diastolic all computational frameworks. Series of diagrams showing blood flow velocity in the aortas of two patients, before and after treatments. Each diagram is labeled with the patient number and treatment stage.

Velocity: End-diastolic velocity streamlines are presented in Figure 5. Post-interventional analysis revealed flow acceleration at the left subclavian artery (LSA) outlet (peak velocity: 1.8 m/s) without endoleak manifestation in both patients. Complete aneurysms flow stagnation was observed, confirming effective AAA exclusion following Castor™ single-branched stent-graft deployment. Virtual embolization simulations demonstrated an inverse relationship between coil length and intra-aneurysmal flow velocity in Patient-1. Notably, Patient-2 exhibited significantly lower velocities within the 3D coil configuration compared to the simpler 2D coil model.

**FIGURE 6:**
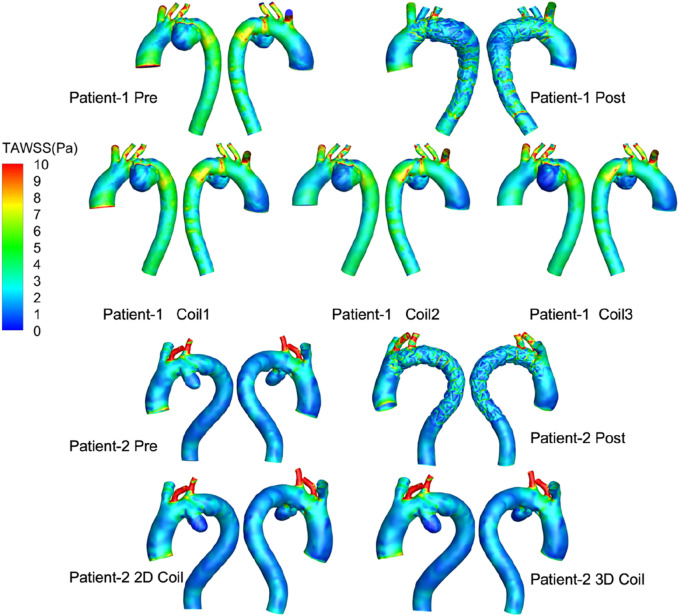
Visualization of blood flow simulations showing total area weighted shear stress (TAWSS) in pascals for two patients. Each patient has pre- and post-procedure models, with additional models using different coil configurations.

TAWSS after surgery is more disordered than before surgery (shown Figure 6), which is mainly caused by stent-graft textile-induced flow separation. As the length of the coil embolization increases, the area of low TAWSS increased significantly in aneurysm, thereby accelerating thrombosis. While low time-averaged wall shear stress (TAWSS <0.4 Pa) is associated with endothelial dysfunction and thrombogenic risk in native vessels, its physiological significance following endovascular repair remains unclear as these shear forces primarily act on the TEVAR device surface. In Patient-1, the low-TAWSS area fraction in the long-coil group (1.24%) substantially exceeded the pre-operative value (0.07%). Consistent with this trend, Patient-2 demonstrated comparable low-TAWSS areas between the 2D (0.64%) and 3D coil configurations (0.66%).

**FIGURE 7:**
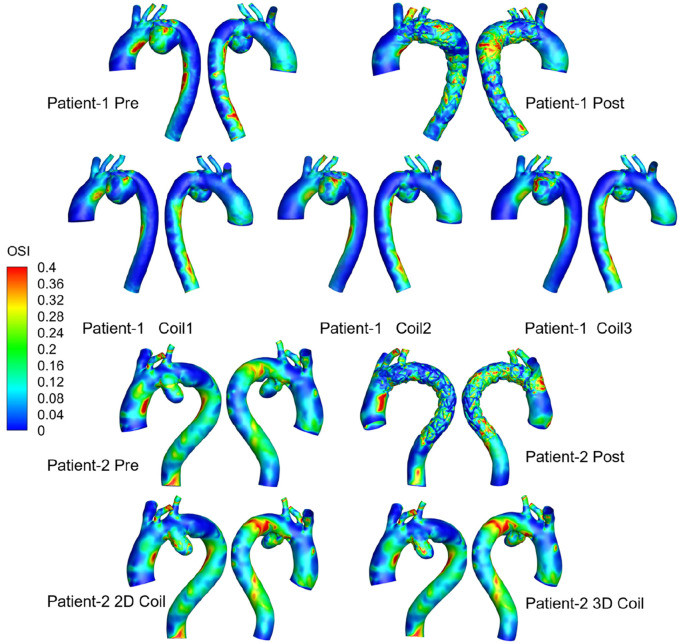
Medical visualization showing three-dimensional models of aorta blood flow for Patients 1 and 2, pre-and post-treatment.

**FIGURE 8:**
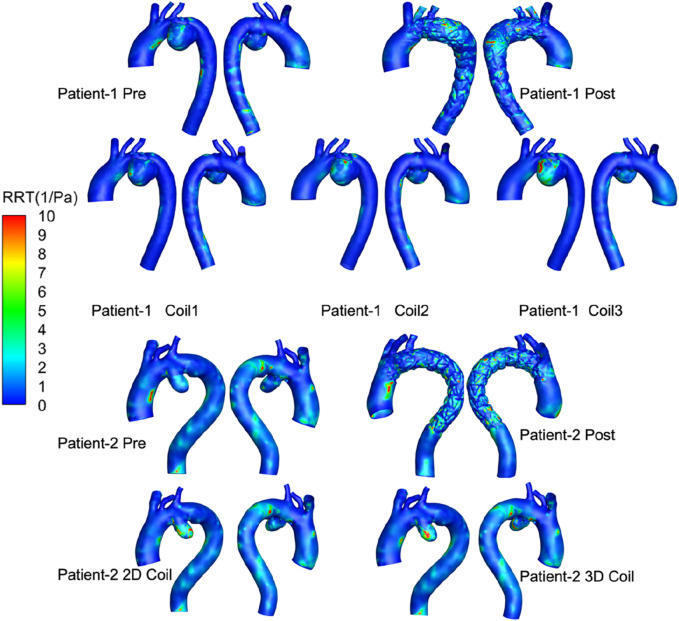
RRT of all computational frameworks. 3D visualizations show aortic regions for two patients, using color mapping to indicate relative residence time (RRT) from 0 to 10 1/Pa. Top row displays Patient-1 pre and post-treatment, and with three coil variations (Coil1, Coil2, Coil3). Bottom row shows Patient-2 pre and post-treatment, with 2D and 3D coil configurations.

The OSI distribution is shown in Figure 7. OSI on the lesser curvature of ascending aorta is significantly higher than that on the greater curvature. The area subjected to high oscillatory shear index (OSI >0.4) was 1.29% for the 3D coil configuration, marginally exceeding the 1.06% observed in the 2D coil model. The RRT results are shown in Figure 8. With the increase in the length of the coil embolization and the increase in the complexity of the 3D coil, the high OSI area and high RRT area are significantly increased. The proportion of luminal surface area exhibiting elevated relative residence time (RRT >10 Pa^−1^) increased with coil length during virtual embolization, rising from 0.14% to 0.31%. Notably, the high-RRT area in the three-dimensional coil configuration (0.31%) represented a twofold increase compared to the pre-operative baseline.

**FIGURE 9:**
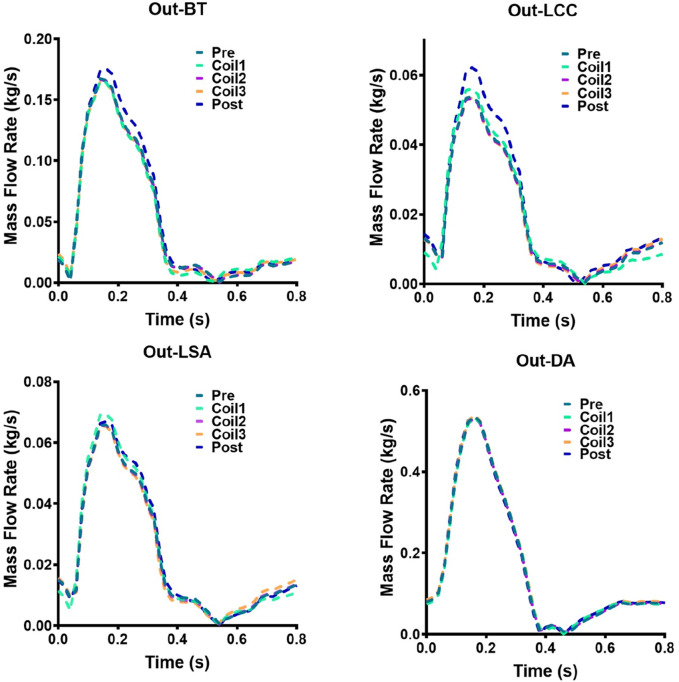
Hemodynamic flow parameters at AAA outflow branches (BT, LCC, LSA, DA) in Patient-1. Four graphs show mass flow rate versus time for Patient-1: Out-BT, Out-LCC, Out-LSA, and Out-DA. Each graph has five lines representing different stages: Pre, Coil1, Coil2, Coil3, and Post.

**FIGURE 10:**
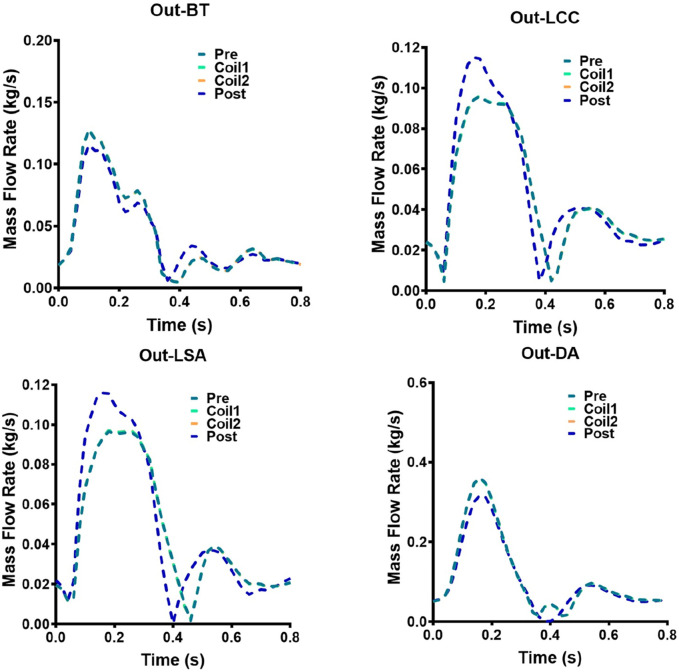
Hemodynamic flow parameters at AAA outflow branches (BT, LCC, LSA, DA) in Patient-2. Four graphs show mass flow rate versus time for Patient-2: Out-BT, Out-LCC, Out-LSA, and Out-DA. Each graph has five lines representing different stages: Pre, Coil1, Coil2, Coil3, and Post.

The mass flow rate of AAA outflow branches (BT, LCC, LSA, DA) of Patient-1 and Patient-2 are shown in Figures 9, 10, respectively. Following Castor™ single-branched stent-graft deployment, both patients exhibited increased LSA flow rates: Patient-1 showed a 6.2% peak systolic flow augmentation (0.0658 vs. 0.0699 kg/s, ΔQ = 0.0041 kg/s, p < 0.05), while Patient-2 demonstrated a more pronounced 20.4% elevation (0.0962 vs. 0.1158 kg/s, p < 0.01), correlating with stent-induced flow acceleration (r = 0.79). The virtual surgery with coil embolization alone had little effect on the flow of the outlets, and the flow of each outlet of the five models basically coincided with that before surgery. Patient-1 had a significant change in the LCC outlet flow, with the maximum mass flow rate increasing from 0.0538 kg/s before surgery to 0.0621 kg/s. Patient-2 had a significant change in the DA outlet flow, with the maximum mass flow rate changing from 0.3606 kg/s to 0.3176 kg/s.

## 4 Discussion

Through virtual coil embolization modeling and hemodynamic analysis, this study evaluated the biomechanical effects of Castor™ single-branched stent-graft in AAA treatment, focusing on pressure distribution, velocity profiles, TAWSS, OSI, RRT, and branch mass flow rates. The key conclusions include: 1) The Castor™ stent-graft effectively isolates the AAA, achieving significant pressure reduction within the aneurysmal sac. 2) No endoleaks were observed post-implantation, with marked flow enhancement in the proximal LCC branch. 3) Coil embolization induces localized hemodynamic alterations without significantly affecting outflow rates at major aortic branches. 4) Increasing coil length and packing density correlates with expanded regions of low TAWSS, elevated OSI, and high RRT values, confirming accelerated thrombus formation within the aneurysm.

The hemodynamic outcomes following Castor™ single-branched stent-graft deployment underscore its efficacy in targeted flow modulation, particularly within complex aortic anatomies. The observed 6.2% (Patient-1) and 20.4% (Patient-2) LSA flow augmentation aligns with the Castor™ single-branched stent-graft design rationale for proximal seal zone optimization, which redirects pulsatile energy toward critical supra-aortic branches (Rong et al., 2022). The stronger correlation between stent-induced flow acceleration and LSA enhancement in Patient-2 may reflect anatomical variations in aortic arch angulation or differential compliance between patients (Sengupta et al., 2022b). Notably, the 11.9% DA flow attenuation in Patient-2, linked to distal landing zone geometry, highlights the delicate balance between branch perfusion preservation and aneurysmal sac depressurization—a critical determinant of long-term AAA stabilization (Li et al., 2023). These findings corroborate the stent-graft’s ability to restore physiological flow hierarchies while introducing measurable spatiotemporal heterogeneity, a trade-off requiring further investigation into endothelial remodeling trajectories (Alagheband et al., 2015; Yao et al., 2022).

While coil embolization has been extensively characterized hemodynamically in intracranial aneurysm management (Wan et al., 2020; Ishii et al., 2021; Fillingham et al., 2023), this study pioneers the first-of-its-kind computational framework for virtual endovascular coiling in aortic pathology. The virtual coil embolization simulations reveal a paradigm of localized hemodynamic control with systemic flow conservation. The minimal branch flow perturbations across five computational models suggest that coil-induced flow modifications operate through microvascular resistance modulation rather than macroscale impedance shifts. The patient-specific flow redistributions—84% LCC variance in Patient-1 *versus* 85% DA localization in Patient-2—may reflect anatomical disparities in collateral network topology or coil packing density gradients. While the preserved mass flow rates support coil embolization’s safety profile, the concomitant TAWSS reduction and OSI/RRT amplification expose a hemodynamic paradox: coils achieve sac thrombosis through low-shear mechanobiology while inadvertently elevating mural stress oscillations (Liu et al., 2022). This duality necessitates optimized coil configuration algorithms balancing thrombogenic efficacy with biomechanical risk mitigation, potentially through machine learning-driven 3D packing simulations that minimize OSI/RRT synergies.

While this study provides a systematic hemodynamic analysis of AAA management via Castor™ single-branched stent grafts combined with coil embolization, several noteworthy limitations warrant consideration: first, the clinical adoption of Castor™ single-branched stent grafts for AAA repair remains underutilized in current practice, resulting in a constrained sample size (n = 2) for this investigation. This small cohort restricts statistical power and may limit the generalizability of hemodynamic patterns to broader AAA morphologies. However, this study has completed hemodynamic analyses for 9 computational cases through 4 virtual procedures, all reconstructed from pre- and post-operative CTA datasets of two patients. Second, although virtual coil embolization accounted for geometric configurations (2D vs. 3D), critical biomechanical factors–including radial support forces during coil deployment and vessel wall compliance changes–were omitted. These simplifications may affect the accuracy of simulated intra-aneurysmal flow reduction and thrombus formation predictions. Third, this analysis focused solely on acute-phase hemodynamic alterations (preoperative, postoperative, and virtual intervention states). Longitudinal follow-up data are essential to validate the durability of flow redistribution effects and long-term aneurysm remodeling. To address the current limitations, prospective multicenter studies with expanded patient cohorts will be conducted to longitudinally monitor hemodynamic profiles under Castor™ single-branched stent graft deployment. This will integrate CFD simulations with serial clinical imaging (e.g., 4D flow MRI and CT angiography) to establish patient-specific hemodynamic risk stratification criteria (Ahmed et al., 2014; Wan et al., 2020). Furthermore, advanced FSI models incorporating coil-vessel biomechanics and artificial intelligence models (Dai et al., 2025) will be developed to refine virtual embolization protocols. These efforts aim to provide a robust evidence base for optimizing endovascular intervention strategies and standardizing Castor™ single-branched stent grafts efficacy evaluation frameworks.

## 5 Conclusion

This study developed the hemodynamic modeling framework for AAA treated with Castor™ single-branched stent grafts. Leveraging patient-specific CTA reconstructions, we established physiologically accurate computational models incorporating 3-element windkessel boundary conditions calibrated with individual hemodynamic profiles. A novel virtual coiling methodology was innovated, enabling morphological simulation of embolization coils with multi-length, multi-configuration deployments in aneurysmal sacs. Through comparative hemodynamic evaluation, we systematically quantified the therapeutic synergies between stent-graft deployment and adjunctive coil embolization.

## Data Availability

The original contributions presented in the study are included in the article/Supplementary Material, further inquiries can be directed to the corresponding authors.
