# Supplementary material for: Hemodynamic modeling of aortic arch aneurysm treatment using the Castor™ branched stent graft: a virtual coil embolization simulation framework
Source: Front Physiol. 2025 Jul 2;16:1629346. doi: 10.3389/fphys.2025.1629346 (PMC12263588; doi:10.3389/fphys.2025.1629346)
Supplement: Supplementary file 2 [file Supplementaryfile1.docx]

- Supplementary Figure S1
- Video 1: supplementary video-Patient-1-40cm
- Video 1: supplementary video-Patient-1-80cm
- Video 1: supplementary video-Patient-1-160cm
- Video 1: supplementary video-Patient-2-3D
